# TH17 cells expressing CD146 are significantly increased in patients with Systemic sclerosis

**DOI:** 10.1038/s41598-019-54132-y

**Published:** 2019-11-27

**Authors:** Amira Gabsi, Xavier Heim, Akram Dlala, Asma Gati, Haifa Sakhri, Ahmed Abidi, Sonia Amri, Bilel Neili, Aurelie S. Leroyer, Alexandrine Bertaud, Monia Smiti Khanfir, Fatma Said, Mohamed Habib Houman, Brigitte Granel, Marcel Blot-Chabaud, Nathalie Bardin, Raja Marrakchi

**Affiliations:** 1 Université de Tunis El Manar, Faculté des Sciences de Tunis, LR05SE05, 2092 Tunis, Tunisia; 2 Aix Marseille Univ, INSERM, INRA, C2VN Marseille, France; 3 Internal medicine service, University hospital center LA RABTA, 1007 Tunis, Tunisia; 4 Université de Tunis El Manar, Faculté de Medicine de Tunis, 1007 Tunis, Tunisia; 5 Service dImmunologie, Pôle de Biologie, Hôpital de la Conception, Assistance Publique-Hôpitaux de Marseille (AP-HM), Marseille, France; 6 Department of Internal Medicine and Therapeutics, Timone Hospital, Marseille, France

**Keywords:** Autoimmunity, Autoimmunity, Autoimmunity, Rheumatic diseases, Rheumatic diseases

## Abstract

Systemic sclerosis (SSc) is an autoimmune disorder characterized by vascular damage, excessive fibrosis and abnormal T cells immune-regulation. CD146 is an adhesion molecule essentially expressed in the vascular system, but also on TH17 lymphocytes. In view of the recently described role of CD146 in SSc, we hypothesized an involvement of CD146 positive TH17 cells in this disease. Compared to healthy controls, we showed that both soluble form of CD146 (sCD146), and IL17A levels were increased in patients with SSc with a positive correlation between both factors. A significant increase in TH17 cells attested by an increase of RORγT, IL17A mRNA and CD4+ IL17A+ cell was observed in patients with SSc. Interestingly, the percentage of TH17 cells expressing CD146 was higher in patients with SSc and inversely correlated with pulmonary fibrosis. *In vitro* experiments showed an augmentation of the percentage of TH17 cells expressing CD146 after cell treatment with sCD146, suggesting that, in patients the increase of this sub-population could be the consequence of the sCD146 increase in serum. In conclusion, TH17 cells expressing CD146 could represent a new component of the adaptive immune response, opening the way for the generation of new tools for the management of SSc.

## Introduction

Systemic sclerosis (SSc) is an autoimmune disease characterized by excessive fibrosis in the skin and internal organs, vascular damage and immune dysfunction. Two major clinical subsets, namely limited cutaneous (lcSSc) and diffuse cutaneous (dcSSc) forms have been described according to the extent of skin fibrosis^1^. Pulmonary fibrosis and pulmonary arterial hypertension (PAH), are the most serious complications and constitute currently the major causes of death^2,3^. The study of new molecular targets is essential in the understanding of the physiopathology of the disease, and the establishment of new therapeutic strategies.

Among them, CD146 has been recently proposed as a new molecular target in SSc^4^. This cell adhesion molecule, also called MUC18 and MCAM, is a membrane glycoprotein belonging to the Immunoglobulin super family^5^. It is ubiquitously distributed on endothelium^6,7^, with a preferential localization at the endothelial junction^7^. CD146 is involved in several functions such as cell adhesion^8^, inflammation^9^, and angiogenesis through different signaling pathways^8^. CD146 also exists as a soluble form (sCD146) in the blood, resulting from the proteolysis of the membrane form^9–11^. In a mice model of SSc induced by bleomycin, we showed that mice lacking CD146 were more susceptible to develop skin fibrosis than wild type animal. Fibrosis development could be prevented by subcutaneous sCD146 injection. We also evidenced the involvement of Wnt pathway since CD146 deficiency was associated with an up-regulation of the canonical Wnt pathway, leading to the profibrotic state^4^.

The expression of CD146 is not restricted to the endothelium since it is also expressed on other cell types such as melanoma cells^12^, extra villous trophoblastic cells^13^ and TH17 cells^14^. TH17 cells are involved in the pathogenesis of several autoimmune diseases including SSc^15^. These cells play a critical role in the pathogenesis of autoimmune diseases, leading to production of several cytokines as IL17A, IL22, IL21 and IL26^14^. The retinoic acid receptor-related-orphan- receptor-gamma T (RORγT), is the key transcription factor required for TH17 cell differentiation and for production of cytokines which participates in protective immunity at boundary tissues^15^. IL17A primarily binds to IL-17RA^16^ and initiates a pro-inflammatory signal, which is strongly involved in the progression of many autoimmune diseases in humans^17,18^.

In SSc, the mRNA and protein expression of IL17A was found to be positively correlated to an outcome index, the modified Rodnan skin score^17,19^.

In view of the role played by CD146 in SSc, we hypothesized a preponderant role of CD146 positive TH17 cells in SSc. To investigate this hypothesis, we explored in the present study, the concentrations of sCD146 and IL17A in serum of patients with SSc as compared to healthy controls. In parallel, we evaluated the proportion of TH17 cells expressing CD146 (CD146+ TH17) over total TH17 cells and analyzed the potential correlations with clinical events. At last, effect of sCD146 was tested on the proportion of CD146+ TH17 among total TH17cells of patients with SSc.

## Materials and Methods

### Ethics statement

All patients gave informed consent and all methods were performed in accordance with the relevant Guidelines and regulations. The Medical Bioethics Committee at the Pasteur Institute of Tunis approved This study under the reference 2015/04/E/FST.

### Blood samples

Peripheral blood samples were obtained from 50 patients with SSc admitted to internal medicine departments of university hospital La RABTA Tunis Tunisia; all patients fulfilled the ACR/EULAR Classification Criteria and were then sub classified according to Leroy *et al*. criteria^1^. The cohort is explained in Table 1. Fifty sera of age and sex matched blood controls (median age 61 (57–70) and sex ratio 10 male/40 female).

**Table 1:** [A] Demographic data and disease characteristics of SSc patients. n: number of subjects.

SSc patients n = 50	Median
Median age (years)	60 (28–68)
Male/female	7/43
Clinical Features	n (%)
Limited cutaneous form	24(48)
Diffuse cutaneous form*	26 (52)
Ulcers, scars, gangrene	17 (65)
Pulmonary fibrosis (PF)	19 (73)
Pulmonary arterial Hypertension (PAH)	20 (76)
Digestive involvement	13 (50)
Kidney involvement	8 (30)
Immunological Results	n (%)
Positive for anti-nuclear antibodies	48 (96)
- centromeric staining (ACA)	24 (47)
- positive for anti-topoisomerase I (anti-topo I)	26 (49)

*According to the classification of ACR 2013.

### Separation of blood cells on a FICOLL density gradient

Peripheral blood mononuclear cells (PBMCs) were prepared by centrifugation over a Ficoll-Hypaque Gradient (Histopaque; Sigma Diagnostics, St Louis, MO) and cryopreseved in 10% dimethyl sulphoxide (DMSO) in fetal bovine serum (FBS). Sera were also collected and stocked in −20° until use.

### Soluble CD146 detection

We measured serum sCD146 concentration using a capture enzyme-linked immunosorbent assay kit (CYQUANT ELISA, Biocytex Marseille, France) according to the manufacturer’s protocol. The ELISA assay was validated previously^4^. A plastic support coated with specific mouse monoclonal anti human CD146 F(ab’)_2_ fragments binds to the sCD146 to be measured.

Next the mouse monoclonal antibody anti-CD146 coupled with peroxidase binds to a remaining free antigenic determinant of the CD146. The bound enzyme peroxidase is then revealed by its activity in a predetermined time on the TMB substrate. After stopping the reaction, the intensity of the signal is directly related to the concentration of sCD146 initially contained in the sample.

### Quantification of IL17A

IL17A was quantified in serum of healthy controls and patients with SSc, using commercially available ELISA kit (Quantikinie ELISA human IL-17A immunoassay R&D Systems France). All the amount of cytokine present in diluted serum will be captured. After several washes to remove the excess, a polyclonal antibody bound to an enzyme specific for human IL17A is added. After washes, the reaction is revealed with a substrate (TMB), giving a color whose intensity is proportional to the amount of IL17A bound and the plates are read at 450 nm and optical density were converted to concentration using a four parametric logistic regression.

### mRNA quantification

#### RNA isolation and cDNA synthesis

Total cellular RNA was extracted from cryopreseved PBMCs using the acid guanidine-phenol- chloroform technique with Trizol (GIBCO, BRL, Bethesda, MD), according to the manufacturer’s instruction. After incubation for 10 min at 65 °C, RNA samples were stored at −80 °C. Total RNA quality and quantity was assessed by absorbance at 260 nm using a nucleic acid and protein analyzer. This RNA was reverse-transcribed in a total volume of 25 µL with 200 units of Moloney murine leukemia virus reverse transcriptase (Invitrogen, US) in the presence of 1.5 µL of 5X First Strand Buffer (Invitrogen, US) (250-mM Tris-HCL; 375-mM KCL; 15-mM MgCL2) in addition to 10 units of RNAsine (Invitrogen, US), 1.25 µL of 100 mM DTT and 1.25 µL of each desoxynucleotide triphosphates (Promega, US) at 10-Mm. After that, all these reagents were incubated at 37 °C for 60 min and then cooled at −20 °C. The integrity and the quantity of the cDNA samples were tested by amplifying β-actin cDNA.

#### Quantitative RT-PCR

Real-time PCR was performed on a The Step-One-Plus Real-Time PCR (Applied Biosystems, CA, USA) using the SYBR Green qPCR Kit (Power Up™ SYBR™ Green Master Mix A25742 Applied Biosystem Thermofischer FRANCE), and continuous fluorescence monitoring. A reaction volume of 25 µl (1.0 µl cDNA) was amplified for 40 cycles after initial denaturation (95 °C, 10 min) with the following parameters: 95 °C for 10 s, 60 °C for 15 s, and 72 °C for 20 s. Samples were run in duplicate, and relative quantification of mRNA level was performed using β-actin as an endogenous reference. We obtained the primer sequences (Table 2) from literature and checked through PCR in silico. We used three primers: RORγT, IL17A and CD146. qPCR data was analyzed by calculating the fold difference individually for each gene. Cycle threshold (Ct) is defined as the number of PCR cycles at which the fluorescence signal rises above the threshold value and is inversely proportional to the amount of template present in the reaction. Ct values of genes and control samples were compared and the fold difference calculated like: Fold CHANGE = 2−ΔCt where ΔCt = CT sample– CT endogenous control. The Ct values were plotted, assuming the same threshold for all genes evaluated. Ct values < 40 were only used for calculation of the PCR efficiency. (This was explained in Step-one-plus book).

**Table 2:** Primer sequences. 1 - βactin (promega). 2- RORδT (eurobio). 3- IL17A A (eurobio). 4- CD146 (eurobio).

Primer		Tm °
β-actin	5′ CATCCTGTCGGCAATGCCAGG 3′ (forward primer) 5′ CTTCTTGGGC ATGGAGTCCTG 3′ (reverse primer)	54
RORδT	5′CTTCCTCAGCGCCCTGTGGTT3′ (forward primer) 5′ CCCAGGACGGTTGGCATTGA 3′ (reverse primer)	68
IL17A	5′ GCAAGAGATCCTGGTCCTGA 3′ (forward primer) 5′ AGCATCTTCTCGACCCTGAA 3′ (reverse primer)	63.8
CD146	5′ CCCGGGCCACCATGGGGCTTCCCA 3′ (forward primer) 5′ GGATCCTCACCGGCTCTCCGGCTC 3′ (reverse primer)	72.4

### Flow cytometry analysis

PBMCs were prepared by centrifugation over a Ficoll technique and cryopserved in 10% SVF- DMSO. Thawed cells were routinely tested for viability by trypan blue exclusion. For cytokine secretion assays, PBMCs were stimulated with complete cell culture medium (Eurobio France) with 1/1500-inonomycine. - 1/2000 PMA - 1/3000 Golgi Stop overnight. Then cells were washed twice with 5% fetal bovine serum (FBS)-phosphate-buffered saline (PBS) prior to staining. First of all, cells were incubated for ten minutes with F_c_ blocking (Human TrueStain FcX^TM^ Ozyme FRANCE) as an isotype control. Then, PBMCs were stained with up to five antibodies for 30 min at room temperature. After staining, the cells were fixed with 2% formaldehyde-PBS prior to analysis on a BD canto flow cytometry (BD Bioscience, San Diego, CA, USA). Lymphocytes were gated based on forward-scatter and side-scatter properties; at least 10,000-gated events were analyzed using the Flow Software (Flow LLC Ashland, Oregon). We calculated absolute cell percentages based on flow cytometry data and complete blood counts. Six colors were used to analyze the surface phenotype and intracellular cytokine production of PBMCs. The antibodies used were CD3 PerCP Cy5/CD4 APC-Cy7/CD25 PE-Cy7/CD146 Alexa fluor 488 for cell surface staining and IL17A PE and FOXP3 APC for intracellular staining and cytokine staining.

Flow cytometry analysis was performed on the canto 2 flow cytometry (BD Bioscience, San Diego, CA, USA). Lymphocytes subsets were gated by first forward versus side light scatter to identify lymphocytes, then CD4+ T cells were gated using a dot plot showing CD3 versus CD4. First of all, TH17 cells were identified using a dot plot showing CD4 and IL17A and then TH17 cells expressing CD146 were gated using a dot plot showing IL17A and CD146. Frequencies of cells were determined using statistical option in FLOWJO V10.5.3.

### *In vitro* sCD146 stimulation on PBMCs from patients with SSc

Fresh PBMCs from patients were used. 5 × 10^5^ cells from each patient were incubated with 100 ng/ml of sCD146 [RPMI-1640 (Gibco, Carlsbad, CA, USA) with 5% AB + human serum, 10 mM HEPES, non-essential amino acids, sodium private, 2 mM L-glutamine (Sigma, St. Louis, MO, USA), 100 units/ml of penicillin and 100 μg/ml of streptomycin (Invitrogen, Carlsbad, CA, USA)] + 100 ng/ml sCD146, then cultured in a humidified incubator at 37 °C with 5% CO2 for 24 hours. For cytokine secretion assays, cells were stimulated overnight with PMA-Ionomycine-Golgi Stop culture medium. And the cells were stained with CD3 PerCP Cy5/CD4 APC-Cy7/CD146 Alexa fluor 488 antibodies for cell surface staining and IL17A PE antibody for cytokine staining.

Flow cytometry analysis was performed on the canto 2 flow cytometry (BD Bioscience, San Diego, CA, USA). Lymphocytes subsets were gated as described above in flow cytometry analysis section.

Finally, using these cells stimulated or not with sCD146, total RNA was extracted and cDNA was synthesized as described above (2.6) and Q-RT PCR was performed using CD146 primers (Table 2).

### Statistical analysis

Results presented as means ± standard deviation. Statistical analyses were performed using Prism (Graph Pad Software, San Diego, CA). A p value < 0.05 was considered statistically significant.

Correlation and linear regression are the most commonly used techniques for quantifying the association between two parameters. The analyses are based on linear relationship between the two variables. A scatter plot is essential before embarking on any correlation-regression analysis to show that this is indeed the case. In our study, correlation and linear regression let us study correlation between sCD146 concentrations, IL17A concentration and TH17 expressing CD146 cells percentages.

## Results

### Epidemiologic study

We included 50 patients with SSc from the internal medicine department of university hospital RABTA Tunis, having a score of 9 or higher according to the ACR/EULAR criteria. Our cohort was composed of 43 women and 7 men and a mean age of the onset of illness equal to 60 ± 16 years [28–68 years]. Rodnan’s score ranged from one to 41 in our cohort and averaged 17. Then, clinical data highlighted several inaugural events such as Raynaud’s syndrome, skin involvement, arthralgia, cutaneous sclerosis, gastro-esophageal reflux. Pulmonary Arterial Hypertension (PAH), is one of the most serious disorders of systemic sclerosis, and is diagnosed by cardiac Doppler ultrasound after exercise dyspnea. Another serious manifestation is pulmonary fibrosis demonstrated by Chest CT examination. Our cohort was constituted by 20 patients with PAH and 19 patients with pulmonary fibrosis (Table 1).

### Soluble CD146 and IL17A concentrations are increased in sera of patients with SSc

**Figure 1:**
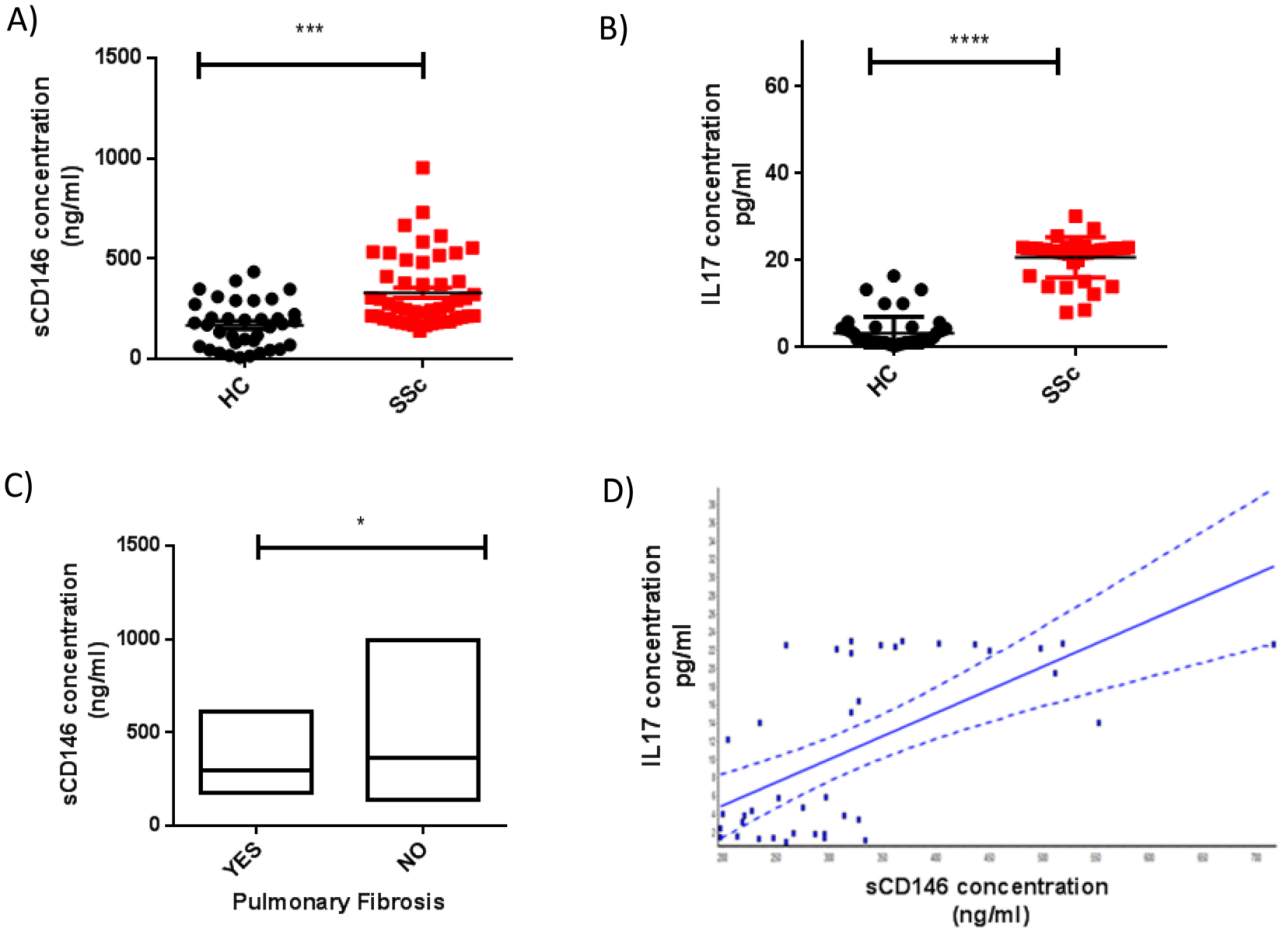
Serum level of soluble CD146 (sCD146) and circulating IL17A in patients with Systemic sclerosis (SSc) and healthy controls (HC). (**A**) Soluble CD146 (sCD146) concentration was significantly higher in patients with SSc (n = 50) than in HC (n = 50) [329.0 ± 24.90 ng/ml *versus* 168.4 ± 19.39 ng/ml, p < 0,0001]. (**B**) The cytokine IL17A concentration was significantly increased in patients with SSc (n = 50) than in (HC) (n = 50) [20.05 ± 1.97 ng/ml *versus* 3.32 ± 0.54 ng/ml, p < 0, 0001]. (**C**) A significant association was observed between pulmonary fibrosis (n = 19) and low levels of sCD146 (p = 0.0147). (Yes = have pulmonary fibrosis/No = have other involvement than pulmonary fibrosis). (**D**) A correlation was observed between the IL17A and soluble CD146 (sCD146) concentrations in patients with SSc (p < 0.0001).

Serum concentrations of sCD146 and IL17A were measured in patients with SSc (n = 50) and compared to age - and sex- matched controls (n = 50). As shown in Fig. 1A,B, sCD146 and IL17A concentrations were significantly higher in patients with SSc as compared to healthy controls (respectively, 329.0 ± 24.90 ng/ml *versus* 168.4 ± 19.39 ng/ml, p < 0, 0001 and 20.05 ± 1.97 ng/ml *versus* 3.32 ± 0.54 ng/ml, p < 0, 0001). Regarding clinical association, we found a significant association between low sCD146 concentrations and pulmonary fibrosis (p = 0.04) as shown in Fig. 1C, while there was no correlation with other clinical disorders described in Table 1. For IL17A, no correlation was observed with clinical disorders. Interestingly, we found a positive correlation between sCD146 and IL17A concentrations (p = <0.0001), as illustrated in Fig. 1D.

### The number of CD146+ TH17 cells is increased in patients with SSc

We analyzed the expression of IL17A and of the master transcription factor of TH17 cells, RORγT, in patients with SSc as compared to control group.

**Figure 2:**
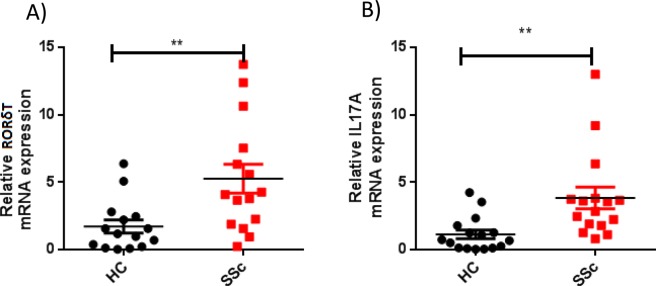
Relative mRNA expression of IL17A and RORγT in PBMCs from patients with Systemic sclerosis as compared to healthy control. (**A**) The expression of the transcription factor RORγT mRNA was higher in patients with SSc than in HC (p = 0, 0013). (**B**) The expression of the cytokine IL17A mRNA was higher in patients with SSc than in HC (p < 0.0001).

The mRNA expression of RORγT and IL17A was first quantified by qRT-PCR in peripheral blood mononuclear cells (PBMCs) of patients with SSc and healthy controls. RORγT and IL17A mRNA expression were significantly higher in patients with SSc as compared to healthy controls (p = 0, 0013 and p = <0.0001) (Fig. 2A,B). For RORγT and IL17A mRNA expression, no significant association was observed with clinical features.

**Figure 3:**
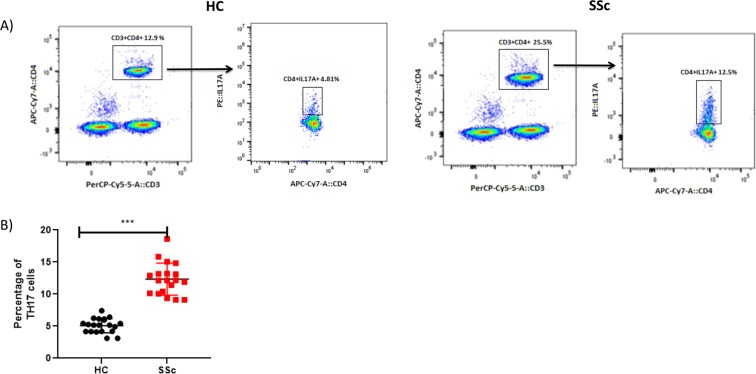
Flow cytometry analysis of TH17 cells in patients with systemic sclerosis as compared to healthy controls. (**A**) Gating strategy of TH17 cells in HC as compared to PBMC of Patients with SSc by FACS: cells were activated with PMA-Ionomycine-Golgi Stop overnight and F_c_ blocking (Humain TrueStain FcX^TM^ Ozyme FRANCE) was used as an isotype control. (**B**) The percentage of TH17 cells in patients with SSc was found to be increased as compared to HC (p = 0.0019).

In a second step, we studied the proportion of TH17 cells over the total CD4+ cells population using a flow cytometry analysis (Fig. 3A). We found a higher percentage of TH17 cells in patients with SSc (Mean 16.95 ± 0.8482), as compared to healthy controls (Mean 9.135 ± 0.2522, p < 0.05) as shown in Fig. 3B.

**Figure 4:**
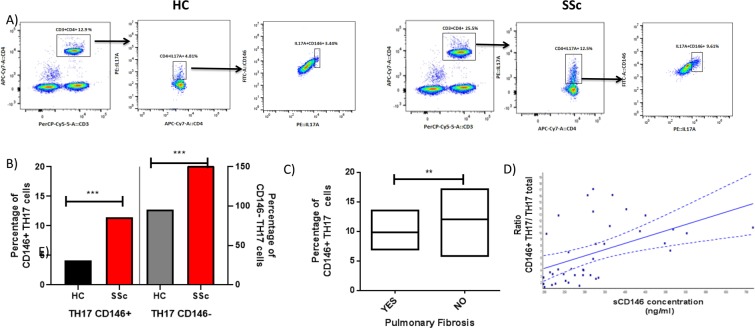
Flow cytometry analysis of TH17 expressing CD146 in Systemic sclerosis. (**A**) Gating strategy for analysis of CD146 expression on TH17 total cells in HC compared to patients with SSc. (**B**) The mean percentage of TH17 cells expressing CD146 (CD146+ TH17cells) and non expressing CD146 (CD146- TH17) among HC and patients with SSc: Percentage of TH17 cells expressing CD146 (CD146 + TH17) is higher in case of SSc than in healthy controls (p = 0.0019). (**C**) The percentage of TH17 cells expressing CD146 (CD146+ TH17) is inversely correlated to pulmonary fibrosis (p = 0.04) (Yes = have pulmonary fibrosis/No = have other involvement than pulmonary fibrosis). (**D**) A correlation is observed between the ratio (CD146+ TH17/TH17 Total) cells and sCD146 concentration in patients with SSc (p = 0.0004).

Then, we analyzed CD146 expression on TH17 cells, we found a higher percentage of TH17 cells expressing CD146 (CD146+ IL17A+ subset) in patients with SSc as compared to healthy controls (Fig. 4A,B). Interestingly the percentage of TH17 cells expressing CD146 was inversely correlated to the pulmonary fibrosis (4 C) but no association with other clinical features was detected.

To better understand the link between TH17 cells and sCD146, the ratio between CD146+ TH17 cells and total TH17 cells was calculated (percentage of TH17 cells expressing CD146/TH17 cells). We found a significant correlation between sCD146 concentration and this ratio (p = 0, 0135), as shown in Fig. 4D.

### sCD146 affects T lymphocytes in PBMCs from patients with SSc

**Figure 5:**
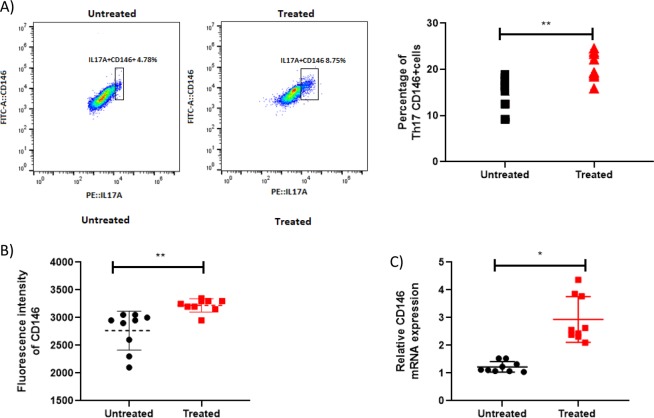
Expression of CD146 on TH17 cells *in vitro*; Expression of CD146 on TH17 cells was analyzed in peripheral blood mononuclear cells (PBMCs) from healthy controls (HC) and patients with SSc. (**A**) Flow cytometry analysis of TH17 cells expressing CD146 in PBMCs from patients with SSc treated with sCD146: in each subpopulation, expression of CD146 on TH17 cells was compared between untreated PBMCs (squares) and treated ones (triangles) in patients with SSc. (**B**) Flow cytometry analysis of TH17 cells expressing CD146 in PBMCs from patients with SSc treated with sCD146: Fluorescence intensity of CD146 is higher on TH17 cells from PBMCs treated with sCD146 compared to untreated ones. (**C**) The expression of the transcription factor RORγT mRNA and IL17A mRNA in PBMCs of patients with SSc before and after treatment with sCD146: there is no difference in RORγT mRNA expression (p = 0.7994) while IL17A mRNA expression was lower after treatment (p = 0.0053). (**D**) CD146 mRNA expression in PBMCs of patients with SSc before and after treatment with sCD146: there is an increase in CD146 mRNA expression after treatment (p = 0, 0232).

We evaluated sCD146 effects on TH17 cells after sCD146 treatment (100 ng/ml for 24 hours) of PBMCs of patients with SSc. Interestingly, an up regulation of the percentage of TH17 cells expressing CD146 (CD146+ IL17A+ subset) was found after sCD146 treatment (Fig. 5A). The number of cells was determined at T0, and after 24 h of sCD146 treatment with 492 million +/− 6088 at T0 and 491 million +/− 6441 at T24, no significant difference was observed (p = 0.8992) attesting for the absence of cell proliferation. In addition expression of CD146 was up regulated after sCD146 treatment as demonstrated by fluorescence intensity analysis (Fig. 5B). Besides, after treatment with sCD146, mRNA expression of CD146 was increased in PBMCs treated with sCD146 as compared to untreated cells (Fig. 5C).

## Discussion

In systemic sclerosis, we recently reported a major role of CD146, a molecule ubiquitously distributed on vascular cells, but also expressed on a subset of lymphocytes involved in the pathogenesis of the disease, TH17 cells. In this study we demonstrated for the time that TH17 lymphocytes expressing CD146 are increased in patients with SSc, suggesting their involvement in the pathology.

In these patients, we found a higher percentage of TH17 cells expressing CD146 and a correlation between sCD146 and IL17A serum concentrations. Interestingly the ratio of TH17 cells expressing CD146/Total TH17 cells in patients with SSc was inversely correlated to pulmonary fibrosis. Finally, *in vitro* treatment of TH17 cells with sCD146 led to an up regulation of CD146 expression. Afterward, PBMCs treatment with sCD146 induced an increase in the mRNA expression of CD146 as compared to untreated cells.

In a previous study, unfavorable evolution of SSc was associated with a decrease of sCD146 concentrations. We recently showed that sCD146 concentrations were increased in patients with SSc and that severe manifestations of the disease, as pulmonary fibrosis, were associated with low sCD146 concentrations^4^. Patients producing high and sustained levels of sCD146 may be protected against the disease complications, accounting for the reduction in the concentration of the soluble form in the case of pulmonary fibrosis^4^. In the present study, we confirm these data, highlighting sCD146 as a novel biomarker for the assessment of the disease. In addition, we evidence a significant correlation between sCD146 and IL17A concentrations. This positive correlation could explain the protective role of sCD146, interfering with the counterbalance of inflammation and fibrosis mediated by IL17A.

In patients with autoimmune disease, an increased proportion of TH17 cells were described in the peripheral circulation and at the sites of active inflammation^20^. Several studies have shown that in SSc, TH17 cells could affect the inflammatory response of the disease, and potentially the fibrosis process^21^. Our results confirm a higher percentage of TH17 cells in patients with SSc and show for the first time that this increase is partially due to a higher percentage of TH17 cells expressing CD146. We propose that in patients, the up-regulation of this sub-population could be related to the sera sCD146 increase. In accordance with this hypothesis, we showed an *in vitro* increase of TH17 cells expressing CD146 after sCD146 treatment. This increase was attributed to an augmentation of CD146 expression since we showed an up-regulation of fluorescence intensity without TH17 proliferation. Such an increase of membrane CD146 expression has been already reported in endothelial cells^22^. We also showed that sCD146 concentrations correlate with the TH17 expressing CD146/TH17 total cells ratio in patients with SSc.

Altogether our results led us to hypothesize that sCD146 could be involved in TH17 cells differentiation and their homing to the site of the disease. Along this line, it has been described that CD146 positive TH17 cells was the sub-population of TH17 cells with an enhanced ability to bind to, and to cross through, vascular endothelium. In addition, CD146 was described as a marker of mouse NK cell maturation^23^. At last, TH17 expressing CD146 are able to migrate from the peripheral circulation to sites of active inflammation^24^. Further studies will be conducted to confirm the role of this sub-population CD146+ TH17 in the fibrotic process, to get more insights into mechanistic pathways and to investigate the potential interest for therapeutic applications.

In conclusion, TH17 cells expressing CD146 could represent a new component of the adaptive immune response and could be potential targets for therapy, opening the way for the generation of new tools for the management of SSc.

## References

[1] van den Hoogen F (2013). 2013 Classification Criteria for Systemic Sclerosis: An American College of Rheumatology/European League Against Rheumatism Collaborative Initiative: ACR/EULAR Classification Criteria for SSc. Arthritis & Rheumatism.

[2] Zhao W (2017). The status of pulmonary fibrosis in systemic sclerosis is associated with IRF5, STAT4, IRAK1, and CTGF polymorphisms. Rheumatol Int.

[3] Chu H (2018). Sirtuin1 Protects against Systemic Sclerosis–related Pulmonary Fibrosis by Decreasing Proinflammatory and Profibrotic Processes. Am J Respir Cell Mol Biol.

[4] Kaspi E (2017). Identification of CD146 as a novel molecular actor involved in systemic sclerosis. Journal of Allergy and Clinical Immunology.

[5] Bardin N, Francès V, Combes V, Sampol J, Dignat-George F (1998). CD146: biosynthesis and production of a soluble form in human cultured endothelial cells. FEBS Letters.

[6] Bardin N (1996). S-Endo 1, a pan-endothelial monoclonal antibody recognizing a novel human endothelial antigen. Tissue Antigens.

[7] Bardin N (2001). Identification of CD146 as a component of the endothelial junction involved in the control of cell-cell cohesion. Blood.

[8] Bardin N (2009). CD146 and its Soluble Form Regulate Monocyte Transendothelial Migration. ATVB.

[9] Kebir A (2010). CD146 Short Isoform Increases the Proangiogenic Potential of Endothelial Progenitor Cells *In Vitro* and *In Vivo*. Circulation Research.

[10] Bardin N (2006). Increased expression of CD146, a new marker of the endothelial junction in active inflammatory bowel disease. Inflammatory Bowel Diseases.

[11] Ito T (2017). Elevated serum levels of soluble CD146 in patients with systemic sclerosis. Clin Rheumatol.

[12] Zheng H, Chen G, DeLouise LA, Lou Z (2010). Detection of the Cancer Marker CD146 Expression in Melanoma Cells with Semiconductor Quantum Dot Label. j biomed nanotechnol.

[13] Kaspi E (2013). Identification of soluble CD146 as a regulator of trophoblast migration: potential role in placental vascular development. Angiogenesis.

[14] Wu C, Goodall JC, Busch R, Gaston JSH (2015). Relationship of CD146 expression to secretion of interleukin (IL)-17, IL-22 and interferon-γ by CD4^+^ T cells in patients with inflammatory arthritis: Cytokine secretion by CD146^+^ CD4^+^ T cells. Clin Exp Immunol.

[15] Castro G (2017). RORγt and RORα signature genes in human TH17 cells. PLoS One.

[16] Gu C, Wu L, Li X (2013). IL-17 family: Cytokines, receptors and signaling. Cytokine.

[17] Bălănescu P, Bălănescu E, Bălănescu A (2017). IL-17 and TH17 cells in systemic sclerosis: a comprehensive review. Romanian Journal of Internal Medicine.

[18] Amatya N, Garg AV, Gaffen SL (2017). IL-17 Signaling: The Yin and the Yang. Trends in Immunology.

[19] Zhou Y (2015). The elevated expression of TH17-related cytokines and receptors is associated with skin lesion severity in early systemic sclerosis. Human Immunology.

[20] Bedoya SK, Lam B, Lau K, Larkin J (2013). TH17 Cells in Immunity and Autoimmunity. Clinical and Developmental Immunology.

[21] Truchetet M-E, Brembilla NC, Montanari E, Allanore Y, Chizzolini C (2011). Increased frequency of circulating Th22 in addition to TH17 and Th2 lymphocytes in systemic sclerosis: association with interstitial lung disease. Arthritis Res Ther.

[22] Bardin N (2003). Soluble CD146, a novel endothelial marker, is increased in physiopathological settings linked to endothelial junctional alteration. Thromb Haemost.

[23] Despoix N (2008). Mouse CD146/MCAM is a marker of natural killer cell maturation. Eur. J. Immunol..

[24] Dagur PK (2011). MCAM-expressing CD4+ T cells in peripheral blood secrete IL-17A and are significantly elevated in inflammatory autoimmune diseases. Journal of Autoimmunity.

